# Comment on Suresh et al. The Short-Term Effects and Tolerability of Low-Viscosity Soluble Fibre on Gastroparesis Patients: A Pilot Clinical Intervention Study. *Nutrients* 2021, *13*, 4298

**DOI:** 10.3390/nu14091836

**Published:** 2022-04-28

**Authors:** J. Wesley Jones, Katrina Lamont, Grace D. Brannan

**Affiliations:** 1 Department of Internal Medicine, Jerry M. Wallace School of Osteopathic Medicine, Campbell University, Buies Creek, NC 27546, USA; katrina.lamont13@gmail.com; 2 Department of Internal Medicine, McLaren Macomb Hospital, Mt. Clemens, MI 48043, USA; grace.brannan@mclaren.org

Zhou and colleagues are commended for their innovative research on the tolerability of “low-viscosity” fibre supplements in symptomatic diabetic gastroparesis patients [1]. Using a randomized controlled crossover pilot clinical study design, they demonstrated the beneficial effects of partially hydrolysed guar gum and gum Arabic on blood glucose regulation with no significant adverse gastrointestinal effects, i.e., comparable to negative controls. Their observations are a major step forward in solving the gastroparesis riddle and challenge the broadly held premise that all dietary fibre should be avoided or minimized in these patients [2].

Furthermore, recent reports have found the treatment efficacy of gastric peroral endoscopic myotomy for gastroparesis to substantially diminish over time [3], and the effectiveness in symptom control with gastric electrical stimulation to be also less than ideal [4]. Importantly, other studies have shown intestinal dysmotility and constipation to be widely prevalent among these patients, and their severity also correlates with gastroparesis symptomatology [5,6,7]. Moreover, one intriguing report demonstrated that healthy volunteers could impair gastric emptying by self-induced constipation [8]. Given these studies, the widely accepted hypothesis that gastroparesis is a primary gastric motor disorder must be reconsidered.

Another clue in solving the gastroparesis riddle are the landmark observations by Burkitt and others who correlated low stool weights with impaired intestinal transit times (ITTs) [9]. For instance, United Kingdom Navy personnel consuming the typical Western low-fibre diet had a mean ITT of 83 h and mean daily stool weights of 104 g. In contrast, rural Ugandan villagers consuming their indigenous high fibre diets had a mean ITT of 36 h and mean daily stool weights of 470 g. In essence, their findings suggest that achieving large bulky stools may restore intestinal motility by reducing ITT, and thereby possibly alleviate or even resolve gastroparesis symptoms. As dietary fibre has weak laxative effects, osmotic laxatives are frequently required to achieve and sustain increased stool bulk (clinical observations by corresponding author).

Based upon these reports and our experience [10], we believe that constipation is a frequently overlooked cause of gastroparesis. Provided this hypothesis is correct, then regardless of the therapeutic intervention employed, sustained symptomatic improvement of gastroparesis would only be expected if stool weights exceed two to three hundred grams daily and if ITTs average less than 40 h. Moreover, ITT could be a non-invasive and inexpensive test to assess intestinal motility. Finally, we suggest that future gastroparesis studies consider stool weights and ITTs as possible variables that may impact study outcomes.
